# Correlation-Based Anomaly Detection in Industrial Control Systems

**DOI:** 10.3390/s23031561

**Published:** 2023-02-01

**Authors:** Zahra Jadidi, Shantanu Pal, Mukhtar Hussain, Kien Nguyen Thanh

**Affiliations:** 1 School of Computer Science, Queensland University of Technology, Brisbane, QLD 4000, Australia; 2 School of Information and Communication Technology, Griffith University, Gold Coast, QLD 4222, Australia; 3 School of Information Technology, Deakin University, Melbourne, VIC 3125, Australia; 4 School of Electrical Engineering and Robotics, Queensland University of Technology, Brisbane, QLD 4000, Australia

**Keywords:** industrial control systems, cyber attacks, anomaly detection, recurrent neural networks, correlation analysis

## Abstract

Industrial Control Systems (ICSs) were initially designed to be operated in an isolated network. However, recently, ICSs have been increasingly connected to the Internet to expand their capability, such as remote management. This interconnectivity of ICSs exposes them to cyber-attacks. At the same time, cyber-attacks in ICS networks are different compared to traditional Information Technology (IT) networks. Cyber attacks on ICSs usually involve a sequence of actions and a multitude of devices. However, current anomaly detection systems only focus on local analysis, which misses the correlation between devices and the progress of attacks over time. As a consequence, they lack an effective way to detect attacks at an entire network scale and predict possible future actions of an attack, which is of significant interest to security analysts to identify the weaknesses of their network and prevent similar attacks in the future. To address these two key issues, this paper presents a system-wide anomaly detection solution using recurrent neural networks combined with correlation analysis techniques. The proposed solution has a two-layer analysis. The first layer targets attack detection, and the second layer analyses the detected attack to predict the next possible attack actions. The main contribution of this paper is the proof of the concept implementation using two real-world ICS datasets, SWaT and Power System Attack. Moreover, we show that the proposed solution effectively detects anomalies and attacks on the scale of the entire ICS network.

## 1. Introduction

Cyber security was not considered in the design of traditional Industrial Control Systems (ICSs) because they were connected to networks isolated from external networks. However, with the interconnection of ICSs and Information Technology (IT) networks to provide remote access for engineers, ICSs are now vulnerable to cyber-attacks. Moreover, cyber-attacks on ICSs intent to disrupt the industrial process can have an adverse impact on critical infrastructures [1]. Hence, there is a growing concern for the cyber-security of ICSs. Many machine learning-based anomaly detection methods were provided to detect cyber attacks carried out against ICSs [2,3,4,5,6,7,8,9]. However, previously proposed machine learning methods do not identify how anomalous behaviour caused by cyber-attacks propagates and damages connected devices in ICSs.

Advanced attacks in ICS networks usually take multiple sequential actions, which may affect different components of a network. Each affected component can be the next target of the attacker. Therefore, to defend against these sophisticated attacks, the attack and the correlated components need to be identified. Existing machine learning-based solutions provide local anomaly detection, and they do not consider other correlated events that can be the source of another attack. This gap is addressed in our paper. In this paper, we present a system-wide anomaly detection method for ICS networks. Our approach is based on a deep learning method, Long Short-Term Memory (LSTM), to identify anomalies and a correlation analysis [10] to identify the interconnected operation of different nodes such that it helps investigate the impact of cyber-attacks from one device to other devices. Furthermore, unlike previous approaches (such as [11]), which are intended to detect anomalous behaviour only, we present a comprehensive system-wide anomaly detection such that it can predict and prevent future steps of an attack. Therefore, the detection results could be useful for containing the impact of a cyber attack on ICSs.

Our work differs from the existing works in many aspects, from a flexible design approach to comprehensive experimental results. In particular, the novelty of the work is bestowing a system-wide anomaly detection method for ICS networks. Furthermore, we intend to couple the recurrent networks with correlation analysis to identify the anomaly’s time and location and subsequently look at correlated devices for signs of attack progress. Finally, the performance of our proposed method is evaluated using datasets generated from real-world ICSs. To the best of the authors’ knowledge, there is no similar research about system-wide anomaly detection in ICS networks. The major contributions of this paper can be summarised as follows:

- We design and develop a novel system-wide anomaly detection method to efficiently manage and control ICS networks. Our design takes advantage of the recurrent neural networks with correlation analysis to effectively detect the anomaly and its potential consequences over the network.
- We provide detailed proof of the concept prototype implementation using real-world data sets to show the effectiveness of our proposed method. We also illustrated the notable findings compared to the existing methods.
- We also point out potential future research directions that need improvement and further investigation based on our findings.

The organisation of this paper is as follows. Background and related works are discussed in Section 2. The architecture of our system-wide anomaly detection method is presented in Section 3. ICS datasets used in this paper are explained in Section 4.1. The evaluation results of our method are presented in Section 4.2. Section 5 provides more discussion and analysis of the results. Finally, we conclude the paper in Section 6.

## 2. Related Works

A common modern architecture of ICSs is comprised of two interconnected subsystems, Operational Technology (OT) and Information Technology (IT) [12]. The OT system consists of Programmable Logic Controllers (PLC), Remote Terminal Units (RTUs), sensors, and actuators responsible for automating industrial processes. On the other hand, the IT system connects geographically dispersed OT systems to the enterprise network for centralised access [2].

Recent years have seen an increasing trend in cyber-attacks targeting ICSs. Consequently, there is a keen interest in developing anomaly detection systems for ICSs [3,13]. However, unlike IT systems, cyber-attacks on ICSs target OT systems to damage the critical infrastructure. Hence, it is wise to monitor the OT system’s process for anomalous behaviour caused by cyber-attacks. Many machine learning-based anomaly detection methods can be found in the literature [2,3,14,15]. It can be noticed that deep learning methods such as LSTM outperform any other machine learning-based anomaly detection approach as the scale of data increases [16,17,18,19]. Therefore, in this paper, we employ an LSTM to identify anomalies in the ICS data.

However, the challenge of applying machine learning algorithms on a high dimensional and low-value density dataset is that highly correlated features may skew the results [10,14,20,21,22]. Su et al. [23] proposed a ‘dynamic data correlation’ approach to improve the performance of machine learning-based anomaly detection methods by identifying the duplicate deployed sensors. Their method is effective against strongly correlated features, for instance, when the coal flow power dips after a small delay in a power plant. However, their method is ineffective on a dataset with weakly correlated features, such as sensors that monitor the process at different stages of a system.

Gottwalt et al. [20] and Kasongo et al. [24] proposed a correlation-based feature selection approach to improve machine learning-based anomaly detection approaches. However, these methods [20,24] are designed to find anomalies in the network traffic. Hence, their approaches [20,24] are not useful in the case of an insider attack on ICSs or anomalous instructions from a compromised engineering workstation to the plant. Moreover, they used correlation analysis only for feature reduction. Instead, we are interested in predicting anomalous behaviour to limit the impact of cyber-attack.

Petladwala et al. [25] proposed another method for an unsupervised anomaly detection approach based on a feature correlation approach. However, their approach is different because they use the canonical correlation method to extract discriminant features (behaviour) in the dataset and then use the clustering method to identify normal and anomalous features. In comparison to Petladwala et al.’s work [25], we are interested in using correlation to predict the impact of a cyber-attack before it completely disrupts the industrial process. Hussain et al. [26] proposed a method that shares a similar motivation to our work but takes a different approach to detect the impact of an attack from one device to other connected devices in the OT system. Their approach has some limitations, (i) it is an offline anomaly detection method and (ii) it requires prior knowledge of the OT systems, which is not always available. Umer et al. [27] proposed an association rule learning-based approach to identify the correlation between different devices in an ICS. However, their approach also relies on expert knowledge to process the data to learn association rules.

Based on the above discussion, the research gap investigated in our paper is the lack of sufficient study about system-wide anomaly detection in the ICS environment. While discussed papers used correlation analysis to select features, we used correlation analysis associated with deep-learning-based anomaly detection to detect anomalies and predict the impact of the anomalies.

## 3. System-Wide Anomaly Detection

**Figure 1:**
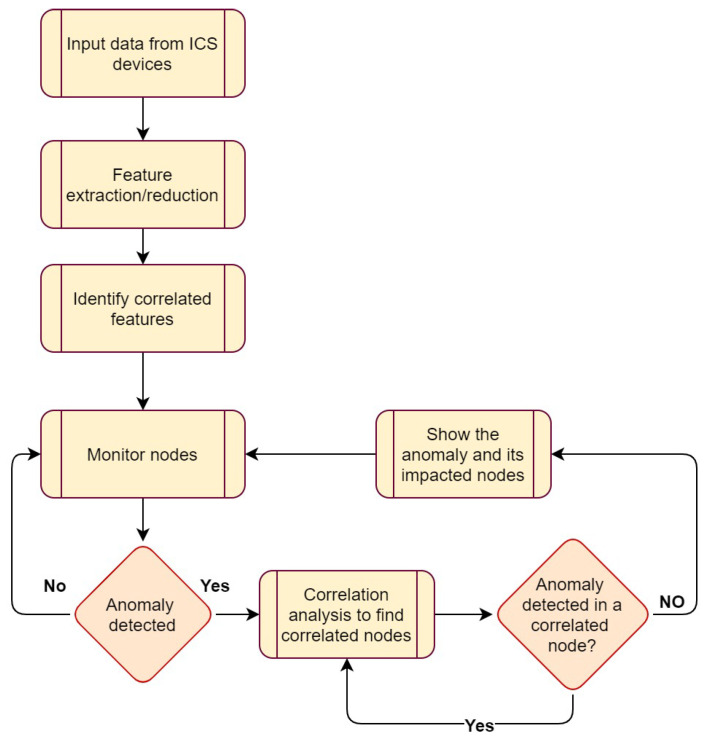
The proposed anomaly detection for ICS networks.

In this section, we will discuss the architecture of our proposed approach for system-wide anomaly detection in an ICS network, as shown in Figure 1.

**Figure 2:**
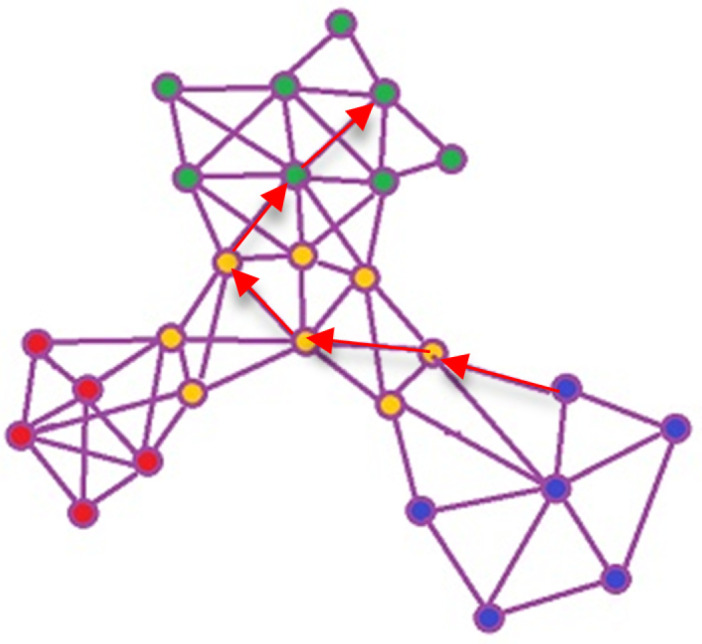
Moving through a network of anomalies.

Different phases of our system-wide anomaly detection method are as follows (as shown in Figure 1):

1. In the first step, the data generated by ICS devices are fed to our system-wide anomaly detection method.
2. The second step is feature extraction/reduction, i.e., to remove duplicate sensors or to isolate key features. For this purpose, correlation analysis of the input dataset is performed. Once the correlation coefficient matrix of all the features is calculated, duplicate features are removed. Two or more features are considered duplicates if the value of correlation coefficients is greater than 0.95.
3. In the third step, a map of correlated nodes is created to identify features/nodes with strong relations with other nodes. Two or more nodes are considered correlated if there exists a positive (greater than 0.5) or a negative correlation (less than −0.5) between their features.
4. In the fourth step, high-priority nodes are identified for monitoring. The network map of the correlated nodes generated in the first phase can be used to identify nodes with the most correlated nodes. These maps help to identify high-priority nodes for monitoring.
5. Anomaly detection can be performed as the key features/nodes are identified. In this phase, we use LSTM models to monitor behavioural patterns of the nodes and detect anomalous changes.
6. Once an anomaly is detected in a node, our proposed method checks all identified correlated nodes to find out whether this anomaly is a part of a big attack. Checking correlated nodes continues until our detection method does not detect more anomalies or all nodes are checked.
7. Finally, the timestamps of the detected anomalies can be analysed to generate a timeline of the detected anomalies. It also helps to visualise the path an anomaly may take in a system (cf. Figure 2).

Our system-wide anomaly detection process model combines deep learning-based anomaly detection and correlation analysis. This approach allows the anomaly detection part to focus on detection, and then the correlation analysis can identify the next target of the attack. Using this process, security analysts will be able to visualise the path an attacker may have taken through a system and identify the devices that have been impacted at the same time or in a series of events. In addition, the information provided by this process can be used to identify attack starting points and the nodes that need further examination or hardening.

Our system-wide anomaly detection model receives features extracted from ICS devices. Initially, it creates a map of all features as nodes. Then, correlation analysis is used to identify correlated features, which are defined as features that have a higher impact on other features and features that are likely to change in clusters. We use this correlation analysis to change the LSTM-based anomaly detection method, i.e., a single feature anomaly detection approach to a system-wide process. This way, anomalies can be tracked comprehensively throughout an ICS. This is the advantage of our method over many detection systems that only monitor individual features or devices. In this paper, we employed the “Pearson correlation method” [28], which is commonly used in the literature to identify correlated features. After identifying correlated nodes, if an anomaly is detected in one node, our method will check the correlated nodes to identify if that anomaly is an isolated anomaly or a part of anomalies that may occur as a chain of events.

**Figure 3:**
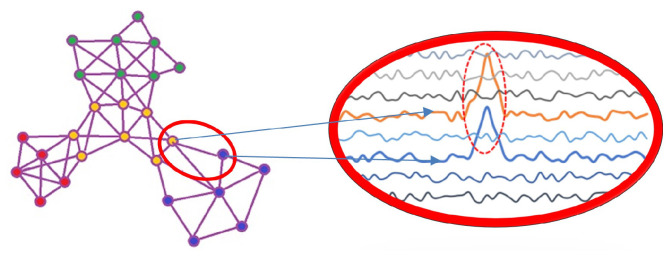
An example of two correlated features.

For anomaly detection in multidimensional datasets, it is recommended to filter out non-correlated features and then train a model with fewer correlated features [14,29,30]. In this regard, a map of correlated nodes is generated to show nodes with the strongest correlated relationship with other nodes. The information about correlated nodes is beneficial for prioritising nodes that should be monitored. Figure 3 shows an example of correlated nodes.

### 3.1. Anomaly Detection

An important aspect in industrial processes, such as power generation and transmission or water treatment plants, is time dependency [31]. For example, the data at the current time point are likely to be related to a previous time point or a time point in the long past, which can be referred to as short-term and long-term time dependency [32]. In this paper, we use the LSTM-based deep learning method for anomaly detection. LSTM is a variation of recurrent neural networks that shows high performance in learning time series data in comparison with other deep learning algorithms [32].

LSTM models are extensively used in different areas where decisions should be made based on the history of inputs. In anomaly detection, this model learns previous network devices’ behavioural patterns and predicts their future behaviour. In this paper, an LSTM-based classifier is used to detect anomalies in ICS devices. This deep neural network method has two outputs identifying normal and abnormal samples. After correlation analysis is performed, LSTM models the features of monitor nodes (aka features). If an anomaly is detected in a node, our system-wide detection method checks the correlated and adjacent nodes. This enables us to identify whether the anomaly is isolated or it is potentially part of a more significant concern.

### 3.2. Correlation Analysis

It is crucial to filter out non-correlated features while working with multidimensional datasets. Some features may appear in a dataset from real-time ICS because two or more devices might represent the same behaviour. This is due to the common practice of recording redundant sensor readings for safety purposes of critical infrastructure automated by ICSs. Hence, a correlation analysis technique would suggest two or more devices are linked in some way to impact each other when they do not. Therefore, we use fewer but highly correlated features that impact each other to train a model [33]. A Pearson correlation coefficient [34], which is employed in this paper, detects statistically significant relationships between two continuous variables where changing a variable may cause a proportional change in the second variable. For example, there is a correlation between two devices. If an anomaly was detected in device 1 initially, the anomaly could have spread to device 2.

Correlation is defined as a term showing a linear relationship between two variables and showing a similarity between the behavioural patterns of these variables [35]. When two variables have a linear relationship, they will have a higher correlation compared with variables with a non-linear relationship.

**Figure 4:**
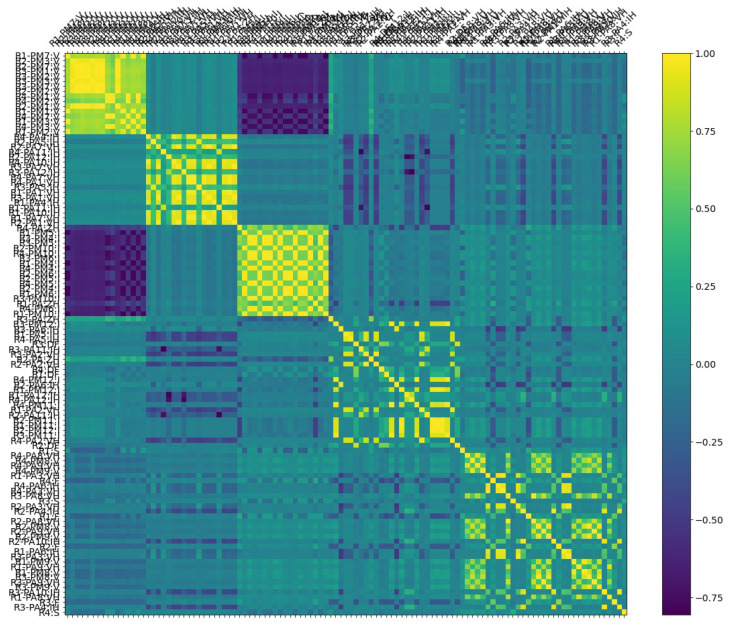
Correlation analysis matrix in Power System line maintenance (The *X*- and *Y*-axes are the same and show Power Systems nodes).

The Pearson correlation method is a method used to find the relevant features using correlation analysis. The correlation coefficient provided by the Pearson correlation method shows the linear connection between two variables. The Pearson correlation coefficient is a number between −1 and 1. A value closer to 0 means a weak correlation. A value approaching 1 shows a positive correlation, and a value approaching −1 indicates a negative correlation. A positive correlation coefficient means an increase in one node causes an increase in another node and vice versa [36]. This correlation values are shown with different colours in Figure 4.

**Figure 5:**
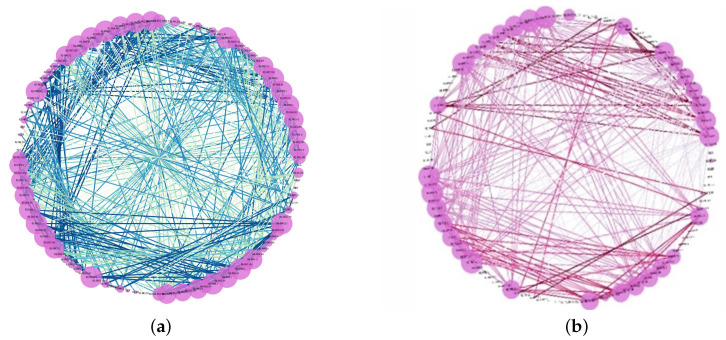
Power systems line maintenance correlation network map: (**a**) positive, and (**b**) negative correlation. The node names in these two figures are the same.

The results of correlation analysis can be represented in a human-readable way using a correlation matrix or correlation map, as shown in Figure 4 and Figure 5. The correlation matrix (cf. Figure 4) shows correlation details in an easy-to-read format for humans, and then the correlation map in Figure 5 shows how many edges we have and what highly correlated nodes are in the network. In the correlation matrix, highly correlated nodes have a darker edge and vice versa.

## 4. Results and Discussion

This section provides the details of two real-world datasets, Power Systems and SWaT datasets, as well as the experimental results of our proposed anomaly detection method.

### 4.1. Datasets

Our proposed system-wide anomaly detection method was tested using two real-world ICS datasets, the Power Systems dataset by Tommy Morris and the Secure Water Treatment (SWaT) dataset by iTrust. We divided these datasets into three subsets, i.e., a training (70%), a validation (10%), and a testing set (20%).

There were not enough publicly available datasets for industrial control systems that can be used for the evaluation of correlation analysis in our paper. However, the datasets used in this paper are the most well-known labelled datasets that provide all the nodes involved in the attacks. These datasets have been collected from two different ICS environments, and this shows that our proposed method can be generalised to other ICS networks with a multitude of correlated devices.

**Power Systems:** The ICS Cyber Attack Power System Datasets were generated by Tommy Morris [37]. It has 37 scenarios that include 28 cases of Attack, 8 cases of Natural Events, and 1 case of No Events. The power system testbed used to generate this dataset consists of power generators, breakers, and Intelligent Electronic Devices (IEDs) that can switch the breakers on or off. There are different features in this dataset available in [37]. Some example features are PA1:VH–PA3:VH (Voltage Phase Angle), PA4:IH–PA6:IH (Current Phase Angle), PA7:VH–PA9:VH (Zero Voltage Phase Angle), and PA10:VH–PA12:VH (Zero Current Phase Angle).

**iTrust SWaT:** The Secure Water Treatment (SWaT) dataset is generated using a scaled-down version of a real-world water treatment plant for the purpose of cybersecurity research [38]. The water treatment plant processes the water using a modern six-stage process. The dataset consists of 35 features. There are 6 types of attacks recorded in the dataset: (1) Attack on a FIT401 sensor, which is a Flow Transmitter that controls the UV de-chlorinator (spoof value from 0.8 to 0.5), (2) Attack on a LIT301 sensor, which is a Level Transmitter (spoof value from 835 to 1024), (3) Attack on P601, an actuator which pumps water from RO permeate tank to raw water tank (switch from OFF to ON), (4) Multi-point Attack (Switch from CLOSE to OPEN (MV201 actuator that is a motorised valve) and OFF to ON (P101 actuator that pumps water from the raw water tank to the second stage)), (5) Attack on MV501 (switch from OPEN to CLOSE), and (6) Attack on P301 (switch from ON to OFF). The P301 actuator in this dataset is a UF feed Pump.

### 4.2. Experimental Results

In this section, we provide two case studies to evaluate the performance of our system-wide anomaly detection method. First, we provide a simple case using the Power System Dataset where anomalies affect multiple devices in the same way. Then a complex case of anomaly detection is provided using the SWaT dataset.

To the best of the authors’ knowledge, there is not any similar work on system-wide anomaly detection in ICS networks that can be compared with our correlation results. However, the performance of our LSTM-only algorithm in detecting local anomalies is compared with other published papers.

#### 4.2.1. Power Systems

This power system line maintenance scenario used to evaluate the performance of our proposed system-wide anomaly detection method includes correlated anomalies such that a number of relays on a specific line were disabled to perform maintenance for that power line [37]. The correlation matrix for different features of this line maintenance scenario in the Power System Datasets is shown in Figure 4. The Pearson correlation method is used to generate the correlation matrix with highly correlated features highlighted using yellow-green. This matrix is also sorted to present related clusters of nodes [14,30].

The identified correlations between the nodes in the matrix (Figure 4) are presented in a human-readable format by converting the correlation matrix to a correlation map, as shown in Figure 5. However, it can be difficult to read a correlation map in the case of a large number of features with many correlations. Therefore, we can split the map into the strongest positive (>0.5, left) and negative (<−0.5, right) correlations to provide meaningful correlation analysis. In the Power Systems dataset, the first node identified with an anomaly was R1-PA4:IH. The nodes shown in Figure 5a,b are the same.

**Figure 6:**
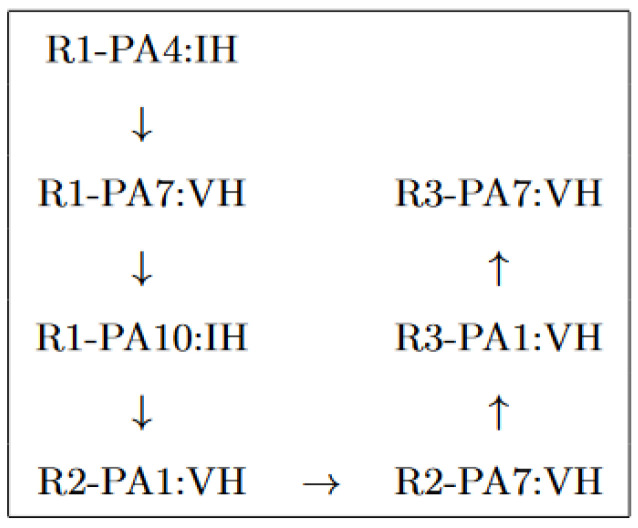
The order of the detected anomalies.

Each newly identified correlated node is checked, and if an anomaly is detected, the correlations of that node are checked until all correlated nodes are checked, or no new anomalies are detected. For R1-PA4:IH, for example, when the model runs, there is a cluster of eight nodes that have detected anomalies in the following order, as shown in Figure 6.

**Figure 7:**
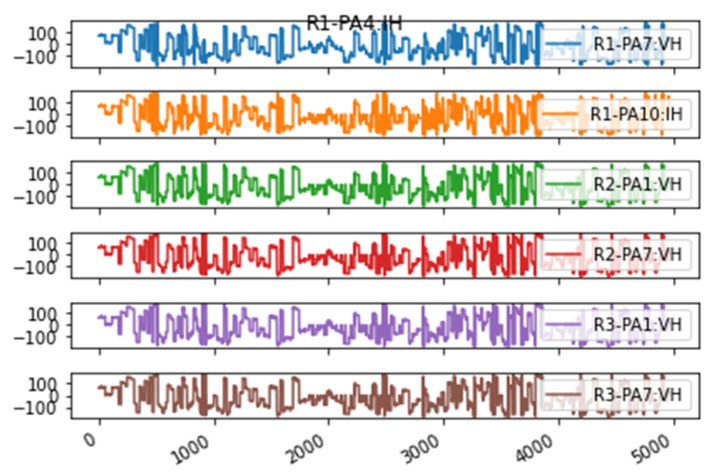
R1-PA4:IH correlated nodes.

Figure 7 shows the unique correlations for the malicious nodes identified. These nodes represent the most correlated features identified by the Pearson correlation method. When an anomaly is detected and its time and location are recorded, that information can then be mapped to show the direction an anomaly took through the system. This is the difference between our solution and other local anomaly detection solutions.

**Figure 8:**
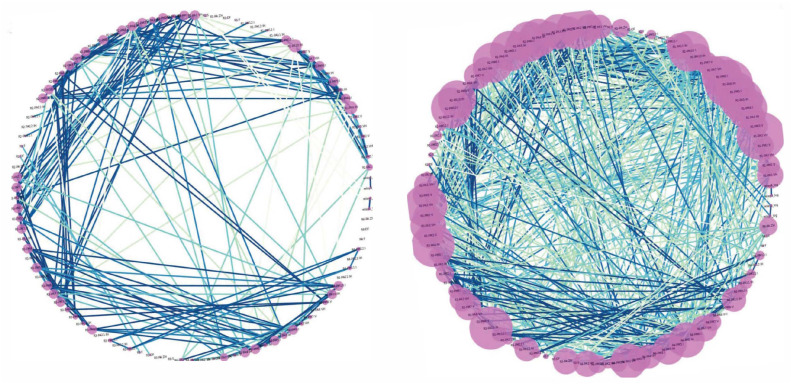
Line maintenance correlation network map with a coefficient of (**left**) 0.95 and greater and (**right**) 0.75 and greater.

In Figure 8, the map on the right shows all correlations with a coefficient of 0.75 and greater, whereas the map on the left is the same information but is limited to a coefficient of 0.95 to show only the strongest correlations and improve visibility. This is helpful when working with datasets that require significant feature reduction.

**Figure 9:**
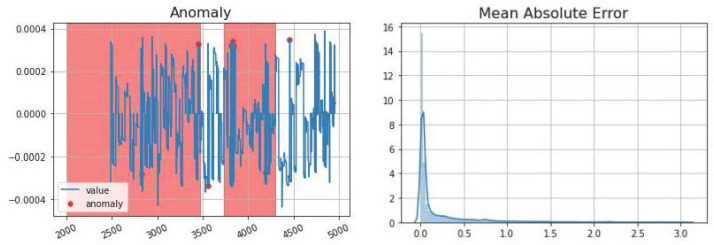
(**left**) R1-PA4:IH detected anomalies, and (**right**) Anomaly detection Mean Absolute Error.

As mentioned earlier, to detect anomalies, we trained the LSTM and used a threshold for Mean Absolute Error (MAE) to determine anomalies. An example of anomalies detected using LSTM in node R1-PA4:IH is highlighted in Figure 9. The performance of this LSTM-only based anomaly detection is shown in Table 1. Once an anomaly is identified in a node (Figure 9), our system-wide anomaly detection method uses correlation analysis to check all other correlated nodes for anomalies. This correlation shows that if an anomaly is detected in a node, the anomaly may spread out from that node. Hence, searching adjacent, correlated nodes can be used to identify all affected nodes in the network [14,30]. The analysis of correlated nodes continues until no further anomalies are detected, as shown in Figure 7. Our proposed method could detect all correlated attacks.

In this paper, we used accuracy, precision, recall, and F1-score as the performance metrics for our LSTM-based anomaly detection method. These metrics are defined by Equations (1)–(4) [39]. Precision represents the percentage of correct anomalies predicted by the anomaly detector. When there is no false positive, the precision is 1.0. Accuracy shows the percentage of correctly classified samples in both anomalous and normal traffic. Recall means the ability of our LSTM algorithm to detect anomalies. When there is no false negative, the recall is 1.0. F1-score is a weighted average of both the precision and recall metrics. These performance metrics are used to evaluate the performance of LSTM-only anomaly detection. These metrics cannot be used to evaluate the correlation analysis.

The results of our LSTM-only based anomaly detection are provided in Table 1. In the Power System dataset, our LSTM anomaly detection provides accuracy and precision of 95%, as shown in Table 1. The results are also compared with other algorithms in other published papers. As is shown, in both datasets, our LSTM-only anomaly detection method provides high performance compared with other algorithms. However, other algorithms in this table are not able to detect correlated steps of multiple-step attacks. They can only detect local attacks without identifying their future correlated actions.

(1) $$Precision=\frac{TruePositive}{TruePositive+FalsePositive}$$

(2) $$Accuracy=\frac{TruePositive+TrueNegative}{TruePositive+TrueNegative+FalsePositive+FalseNegative}$$

(3) $$Recall=\frac{TruePositive}{TruePositive+FalseNegative}$$

(4) $$F_{1}=2\times \frac{precision\times recall}{precision+recall}$$

While advanced cyber-attacks in ICS environments use multiple steps and affect different devices to reach their final target, existing methods do not analyse the impact of the detected attacks on other devices [40,41,42,43]. The contribution of our paper is the combination of high-performance anomaly detection with correlation analysis. Once the LSTM algorithm detects an anomaly in a node, our proposed method checks all identified correlated nodes to find out whether this anomaly is part of a big attack. The ground truth for the correlation between devices was manually extracted from each dataset. The detailed information on simulated testbeds in SWaT and Power System Datasets and the interactions between devices in each attack is available in [38] and [37], respectively. Using the information of device interactions and the timestamp of attack samples provided in [37,38], we could obtain the ground truth of the correlation interactions. For all detected anomalies our proposed method could successfully detect all correlated anomalies.

**Table 1:** Evaluation results of our LSTM-based anomaly detection.

Dataset	Method	Precision	Recall	F1	Accuracy
Power System	Our LSTM-only algorithm	0.95	0.96	0.96	0.95
Power System	K-nearest Neighbours [44]	0.88	0.91	0.90	0.85
Power System	Adaptive Boosting [44]	0.73	0.96	0.83	0.72
SWAT	Our LSTM-only algorithm	0.96	0.90	0.93	0.97
SWAT	SVM [45]	0.93	0.70	0.80	…
SWAT	1D CNN combined records [45]	0.97	0.79	0.87	…
SWAT	1D CNN ensembled records [45]	0.87	0.85	0.86	…

#### 4.2.2. iTrust SWaT

**Figure 10:**
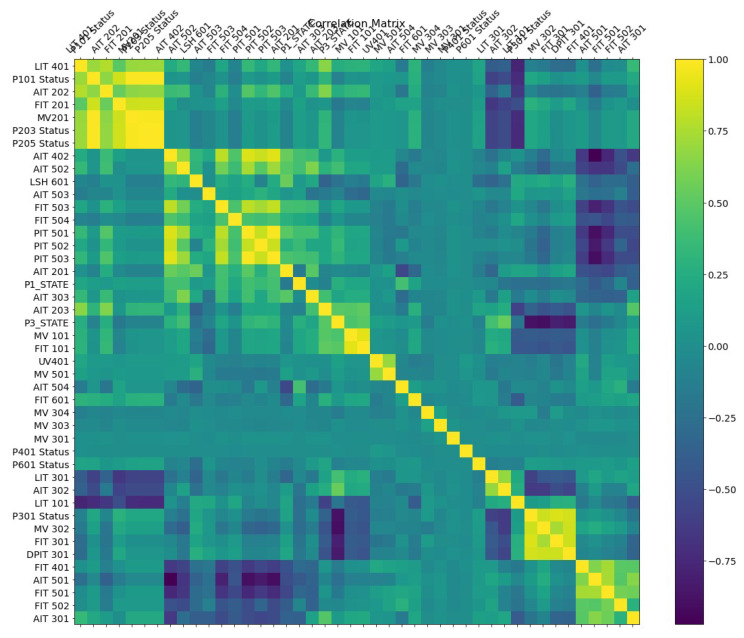
SWaT correlation matrix.

**Figure 11:**
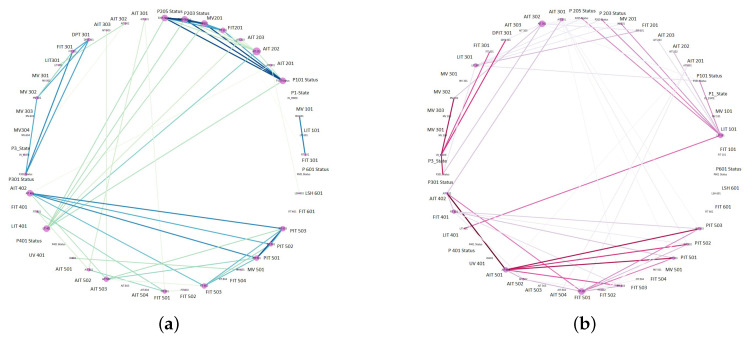
SWaT positive and negative correlations: (**a**) Positive (>0.5), (**b**) Negative (<−0.5).

The second scenario used to evaluate the performance of our system-wide anomaly detection is based on the SWAT dataset. The correlations between different features of the SWAT dataset are represented using a correlation matrix, as shown in Figure 10. Using a correlation matrix (Figure 10), we generated the two maps in Figure 11 for the strongest positive (>0.5) and negative (<−0.5) correlations. A positive correlation means a relationship that when one variable increases, another variable also increases. On the other hand, a negative correlation shows variables that change in opposite directions [14,30].

The maps in Figure 11 can be used to find the high-priority nodes based on strong correlations. For example, nodes AIT402 and LIT401 are in strong positive correlation with other nodes. Meanwhile, nodes FIT401, AIT501 and LIT101 are in strong negative correlations with other nodes. Hence, any change in these nodes may impact the entire network, and hence they are critical to be monitored.

The first attack in the SWAT dataset targets water flow sensor FIT401 by spoofing values from 0.8 to 0.5, which leads to the shutdown of the de-chlorination process by switching off UV401.

**Figure 12:**
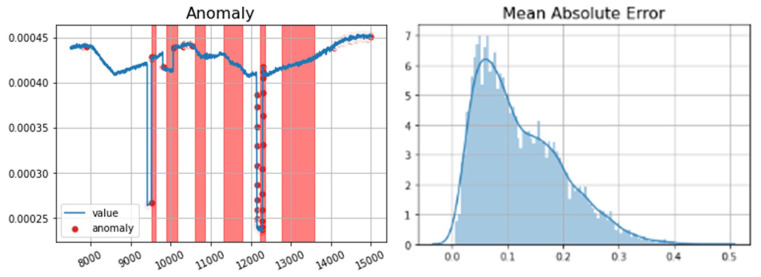
(**left**) FIT401-detected anomalies, (**right**) Anomaly detection errors.

Figure 12 shows the result of anomaly detection using LSTM for node FIT401 with the ground truth highlighted in red. An anomaly detected in the overlapping red areas (ground truth) is a true positive, and an anomaly found outside the red areas is a false positive. In the SWaT dataset, our LSTM-only algorithm provides an accuracy of 97% and a precision of 96%. The performance metrics are provided in Table 1. For each detected anomaly, our correlation analysis could identify all correlated devices affected by the anomaly. The correlated devices were compared with the ground truth manually extracted from the SWaT dataset. All correlated devices were correctly detected in our proposed method.

## 5. Analysis of Findings and Future Work

**Figure 13:**
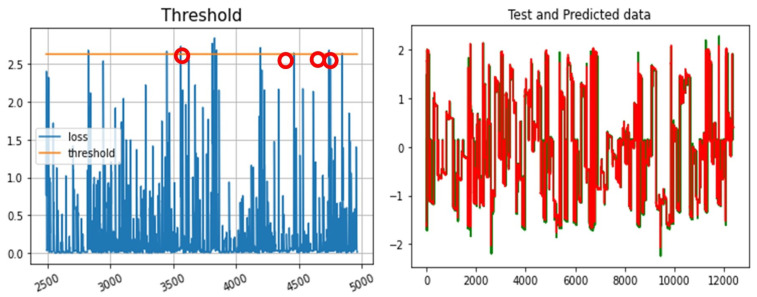
(**left**) Detection threshold for Power System Dataset, (**right**) Prediction of R1-PA4:IH values using LSTM (predicted values (red) and the actual values (green)).

The Power Systems dataset provides various types of events that can be used for anomaly detection analysis. For example, for R1-PA4:IH, there are 10 detected anomalies [14,30]. For each detected anomaly, the LSTM method predicts the expected behaviour of the node (Figure 13 right), and then, a threshold value (Figure 13 left) helps to identify the anomaly area.

The rationale for using this LSTM model for anomaly detection in time-series data is that we can train this model with normal behavioural patterns. Then, we can test the testing output to determine when the model encounters anomalous data that is outside the norm. A threshold value is used to identify anomalous data.

In this paper, the anomaly threshold is selected as 10% to detect anomalies. As shown in Figure 13 left, anomalies are located above this threshold. LSTM learns the normal behaviour in training, and then, it will be able to predict the expected future behaviour in the testing datasets. Our LSTM model was able to effectively predict values closely aligned with the testing data, as shown in Figure 13 right. Deviations from these predicted values will be anomalies.

**Figure 14:**
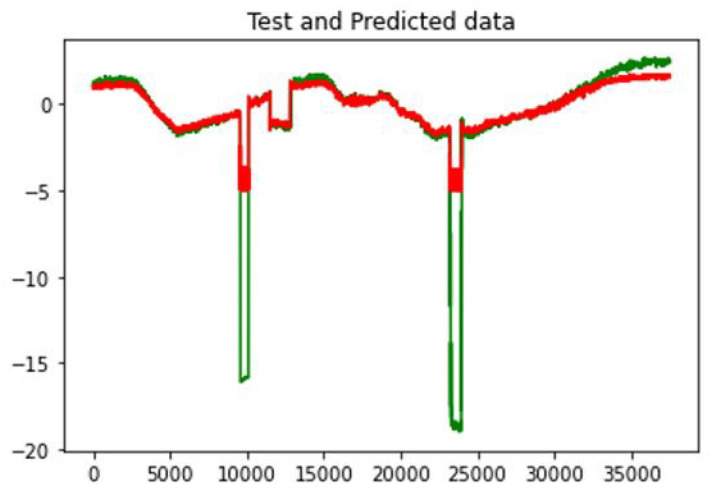
Testing and predicted data for FIT401 (predicted (red) and the actual values (green)).

For the SWaT dataset, a similar threshold value of 10% was set to show the differences between predicted and recorded values as normal and anomalous. Figure 14 shows the testing and predicted values for the FIT401 sensor in this dataset. The accuracy of our LSTM-based anomaly detection in the Power System dataset was 95%, and it had 97% accuracy in the SWaT dataset.

While current anomaly detection methods are designed to monitor individual ICS devices, advanced ICS attacks have multiple steps, and existing anomaly detection methods do not investigate the correlation between anomalies to detect larger anomalies. Our system-wide anomaly detection method is able to identify anomalies with high accuracy in both Power System and SWaT datasets. The benefit of our method is it could detect correlated anomalies as well.

The results in the power systems example show that the model is capable of detecting anomalies. It also shows that the correlation analysis performed preemptively can check correlated nodes and perform system-wide anomaly detection quickly without the need to reassess correlation every time an anomaly is detected.

The correlation analysis does not need to be performed ahead of time. The first time correlation analysis is performed, it is to aid in feature analysis and selection. It would also be possible to perform correlation analysis every time an anomaly is detected. This can slow down detection but has the added benefit of allowing new correlations to be discovered. If there was a new attack that moved differently through nodes, running correlation analysis again when a new anomaly is detected would make it easier to track and identify attack paths. This approach has potential, especially if the anomaly is caught late and there are anomalies before this one, as correlation analysis shows a strong correlation. Once a single anomaly is detected, it can systematically examine each correlated node. The performance results in Table 1 showed that our LSTM algorithm could detect 95% of the attacks in Power System and 97% of the attacks in Swat datasets. Then, correlation analysis could detect 100% of the nodes affected by these identified anomalies. Our system-wide anomaly detector, made from anomaly detection and correlation analysis, is capable of detecting different actions of a multi-step attack that interact with each other. The results of the experiments have validated the proposed approach. However, due to the lack of similar work, we were not able to compare the performance of the entire system-wide anomaly detection with other papers.

While we have shown that LSTMs are effective, there is much potential for future work in this area. Other variants of LSTMs, such as the number of layers and parameters of layers, can be automatically searched to optimise the performance. Other architectures of recurrent neural networks, such as Gated Recurrent Units (GRUs) with fewer parameters, can also be examined. Specifically, the recent Transformer architecture with a strong capability for multimodal data could help to collaborate better features coming from different sources and nature. Experimenting with new architectures will further validate the proposed approach across multiple networks to search for further optimal detection and prediction performance.

## 6. Conclusions

Advanced attacks in industrial networks are professionally designed. They take advantage of the weak security of ICS networks and gain access to an ICS device. Then, they will be able to move into the network and affect other devices. Therefore, it is crucial to create a graph of action and discover the devices affected by the attack. Each affected device can be the source of another attack. Despite existing anomaly detection methods that are not able to follow attack actions, in this paper, we proposed the use of a combination of deep learning-based anomaly detection with correlation analysis to expand local anomaly detection to system-wide anomaly detection. The results showed that our proposed method could detect anomalies accurately and then check adjacent and correlated nodes for other anomalies. Furthermore, the proposed method can predict the future set of events in the case of an ICS attack by identifying other nodes correlated to the node with anomalous behaviour, which is immensely significant in an ICS network. We used two real-world datasets to evaluate our bestowed method, and the results showed high accuracy in both datasets. Note this paper did not consider attacks such as advanced persistent threats (APT). These APT attacks take multiple malicious actions in different based cyber kill chain phases. Finding the correlation between these actions will help to detect these advanced attacks in earlier phases and prevent the attack against the final target. In the future, we intend to explore how correlation analysis can help to detect APT attacks. However, we leave this for future work. In the aspect of computational complexity, our proposed framework needs more computation as all nodes are monitored for anomaly detection purposes, and in the case when an anomaly is detected, all adjacent nodes should be analysed. However, this computational complexity was not investigated in this paper, and it will be studied in our future work.

## Data Availability

Not applicable.
